# In-Flight Transmission of a SARS-CoV-2 Lineage B.1.617.2 Harbouring the Rare S:E484Q Immune Escape Mutation

**DOI:** 10.3390/v14030504

**Published:** 2022-02-28

**Authors:** Mats Martinell, Tanja Andersson, Steinar Smørholm Mannsverk, Julia Bergholm, Patrik Ellström, Anna Hill, Johan Lindh, Rene Kaden

**Affiliations:** 1 Primary Care and Health, Uppsala County Council, 75320 Uppsala, Sweden; mats.martinell@regionuppsala.se (M.M.); tanja.malin.linnea.andersson@regionuppsala.se (T.A.); 2 Department of Public Health and Caring Sciences, Uppsala University, 75237 Uppsala, Sweden; 3 Clinical Microbiology and Hospital Hygiene, Uppsala University Hospital, 75237 Uppsala, Sweden; steinar.smorholm.mannsverk@akademiska.se (S.S.M.); julia.bergholm@akademiska.se (J.B.); patrik.ellstrom@medsci.uu.se (P.E.); anna.hill@akademiska.se (A.H.); johan.lindh@akademiska.se (J.L.); 4 Department of Medical Sciences, Clinical Microbiology, Uppsala University, 75236 Uppsala, Sweden; 5 Science for Life Laboratory, Clinical Genomics Uppsala, 75237 Uppsala, Sweden

**Keywords:** SARS-CoV-2, delta, E484Q

## Abstract

We describe a flight-associated infection scenario of seven individuals with a B.1.617.2 (Delta) lineage, harbouring an S:E484Q point mutation. In Sweden, at least 10% of all positive SARS-CoV-2 samples were sequenced in each county; the B.1.717.2 + S:E484Q combination was not detected in Sweden before and was imported within the scenario described in this report. The high transmission rate of the delta lineage combined with the S:E484Q mutation, associated with immune escape in other lineages, makes this specific genetic combination a possible threat to the global fight against the COVID-19 pandemic. Even within the Omicron wave, the B.1.617.2 + S:E484Q variant appeared in community samples in Sweden, as it seems that this combination has an evolutionary gain compared to other B.1.617.2 lineages. The here described genomic combination was not detectable with the common fasta file-based Pango-lineage analysis, hence increasing the probability of the true global prevalence to be higher.

## 1. Introduction

The World Health Organization (WHO) labels variants that have an impact on diagnostics, treatment (or vaccines), evidence of increased transmissibility, and evidence of increased disease severity as variants of concern (VOC) [1]. Mutations that either increase transmissibility or render immune escape typically result in an evolutionary gain and might quickly become dominant. The global prevalence of SARS-CoV-2, results in a high number of mutations, even though the mutation rate of the virus itself is considered to be low (0.1 per genome per infection cycle) [2]. The B.1.617.2 (Delta) lineage emerged in India during the spring of 2021 and has since become one dominant variant globally [3]. It outcompeted the former predominant lineages B.1.1.7 (Alpha) and B.1.617.1 (Kappa). The highest global prevalence of B.1.617.2 was observed in the middle of June 2021.

The lineage B.1.617.2 is linked to reduced sensitivity to neutralizing antibodies due to a L452R substitution [4]. Another mutation, which is common in the Kappa lineage but not in the Delta variant, causes the substitution of glutamic acid to glutamine at location 484 (S:E484Q) in the receptor binding domain of the spike protein. This substitution is also associated with decreased antibody neutralisation effect [5].

The combination of E484Q with the B.1.617.2 lineage has the potential to be an evolutionary gain for SARS-CoV-2 [6]. Due to the risk of a higher transmission potential, the combination of B.1.617.2 with E484Q was added to the list of variants under monitoring (VOM) in August 2021.

As of 16 August 2021, more than 300,000 sequences of the lineage B.1.617.2 were available on GISAID [7]. Only 401 (0.13%) of them harboured the immune escape mutation E484Q, first reported on 30 January 2021 in the USA [8]. However, on 23 August, the E484Q mutation was found in 509 sequences representing a 27% increase in only a week’s time. Even though the E484Q mutation was found in other lineages (B.1.617.1, B.1.617.3), it was the B.1.617.2 lineage that became dominant [8].

Regarding the reported cases of this combination (709), no significant increase was observed through the end of February 2022.

In this report, we present genomic surveillance combined with clinical contact tracing of a highly transmissible and vaccine-evading chain of infections with B.1.617.2 harbouring S:E484Q that resulted in an uncharacteristically high prevalence of spinning vertigo, a possible neurotrophic symptom. The transmission was linked to a commercial flight from Faro, Portugal to Stockholm, Sweden.

The literature disagrees as to whether air travel is a risk or not, regarding COVID-19 transmission [9]. As different lineages have different transmissibility, a risk assessment must be conducted for each lineage and each combination. On its own, the Delta lineage consists of 232 AY- sublineages, and each of them has specific mutations, which might cause differences in characteristics, such as transmissibility and virulence.

## 2. Materials and Methods

### 2.1. Background and Contact Tracing

Uppsala is a county in Sweden with over 376,000 inhabitants. COVID-19 testing and contact tracing within the county are organized by the Uppsala County Council. COVID-19 is classified as a notifiable disease under the Communicable Diseases Act (Swedish judiciary code 1988:1473); therefore, every case (index) is subjected to contact tracing. After testing positive, every index is contacted within 24 h by a contact tracer who collects information on where, how, and from whom the index was most likely infected.

### 2.2. Genome Sequencing and Assembly

All SARS-CoV-2 tests in Uppsala County are analysed at the laboratory of Clinical Microbiology and Hospital Hygiene, Uppsala University Hospital, with validated methods according to national and international healthcare standards. RNA from patient nasopharyngeal and pharyngeal swab samples was extracted with a Chemagic 360 (Perkin Elmer, Waltham, MA, USA), according to the manufacturer’s instructions. All samples that are positive for SARS-CoV-2 with a cq value below 30 are subjected to sequencing. Sequencing-library preparation, including cDNA synthesis, PCR, end-preparation, barcoding, and adapter ligation was conducted according to the ARTIC protocol [10]. Sequencing and base calling (Parameter: super accurate basecalling) were performed on a GridION sequencer (Oxford Nanopore Technologies, Oxford, UK), with its accompanying MinKNOW software (version 4.3.24). Sequence raw data were imported and assembled and analysed in Geneious Prime (version 2021.1.1) [11] through an in-house developed bioinformatic workflow. The BBDuk plugin (Biomatters inc., Newark, NJ, USA) was used for adapter and quality trimming. Read mapping against the SARS-CoV-2 reference sequence NC_045512.2 was performed using the Minimap2 plugin (version 2.17) [12]. The consensus sequence required at least 4 reads and a base-calling frequency of >50%. The sequences had a genome coverage of 93-1.696.

### 2.3. Sequence Analysis

The consensus sequences were analysed on the web-based Pango Lineage Assigner (accessed on 19 August 2021) [13]. Mutational analysis was performed by applying an in-house designed library of nucleotide annotations in Geneious Prime, targeting SARS-CoV-2 VOC and VOI-defining mutations. The relevant mutations were extracted from outbreak.info, accessed on 24 February 2022 and Covariants.org, accessed on 24 February 2022 [8,14]. The described workflow up to this point was validated and was externally proven with international quality assessments, and the sequences, which were obtained, were included in the Swedish national COVID-19 surveillance.

In addition to the validated workflow, phylogenetic analysis was performed by aligning the sequences using the MAFFT alignment algorithm [15]. A phylogenetic tree from the alignment was constructed using the Geneious Tree Builder, and a consensus tree was generated with the Tamura-Nei genetic distance model, Neighbour-Joining method with 1000 bootstrap replicates, and a clade support threshold of 50%. The phylogenetic tree was subsequently annotated using the Interactive Tree of Life web-based tool [16]. All sequences are available from the Gisaid database.

## 3. Results

### 3.1. Case Descriptions and Flight Scenario

The aircraft (Airbus A320-251N, 174 seats) departed from Faro, Portugal in the first week of August 2021 and arrived at Stockholm airport, Sweden after a flight time of approximately four hours. It is always mandatory to wear facial masks during the flight, except during meals. Table 2 lists the characteristics of all cases (indexes). Index 1 and 2 were a couple traveling together, while index 3 was a member of the cabin crew. Index 3 did not converse with the couple (index 1 and 2) and kept his facial mask on during the flight. Index 4 was a customs officer on duty, in a section of the airport terminal where travellers pass by after disembarking. The couple (index 1 and 2) passed through this area without stopping but did not wear facial masks during this time. Index 5 was the cabin crew member’s wife, who was in close contact with him from the day he landed until he developed symptoms. Index 6 and 7 were two travellers from Malaga, Spain who landed at the same airport, within 24 h prior to the Faro flight from Portugal. Index 1–7 all tested PCR positive for SARS-CoV-2 in the following days. We were not able to directly link index 1–5 with index 6 and 7, but both clusters had in common that the passengers arrived from the southern Iberian Peninsula. A family of four, seated seven rows behind the couple (index 1 and 2) on the same flight from Faro, Portugal, also tested PCR or antigen positive for SARS-CoV-2 after the flight. However, their samples were not attainable as they reside outside Uppsala County.

### 3.2. Clinical Picture and Vaccine Status

The couple traveling together (index 1 and 2) developed symptoms on day one and two after the flight, respectively (Table 2). Backward contact tracing did not reveal any obvious source of infection. Both tested PCR positive two days after the flight, and both were partially vaccinated against COVID-19 (Table 2). The cabin crew member (index 3) developed symptoms and tested PCR positive three days after the flight and was fully vaccinated (Table 2). Index 3 left Stockholm and returned from Faro to Stockholm on the same day. During the flight back to Stockholm, he left the cockpit and stood within two meters from index 1 and 2 for more than 15 min. His wife (index 5) developed symptoms eight days after he returned home and was also fully vaccinated (Table 2). The customs officer (index 4) developed symptoms two days after the flight, tested PCR positive 3 days after the flight, and received the first vaccine dose six days prior to the flight (Table 2). Index 6 developed symptoms two days after the flight from Malaga, Spain and was fully vaccinated (Table 2). Index 7 tested PCR positive for SARS-CoV-2 six days after the Malaga flight but no further information regarding symptoms and vaccination status was available. The cabin crew member’s (index 3) daughter (received the first dose of Pfizer) and granddaughter visited his house in the days after the flight and later fell ill and tested PCR positive for SARS-CoV-2.

### 3.3. Sequencing Results

**Figure 1:**
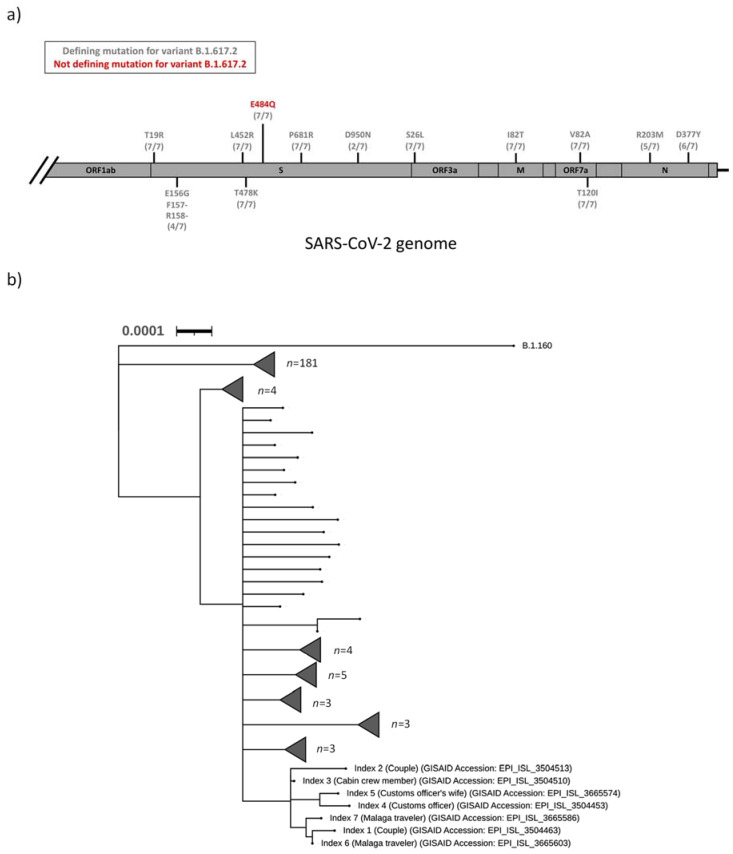
VOC and VOI-defining mutations detected in the SARS-CoV-2 sequences of the indexes and phylogenetic analysis of all sequenced SARS-CoV-2 samples in Uppsala County, during the first two weeks of August 2021. (**a**) Defining mutations detected in the samples of index 1-7. The SARS-CoV-2 genome is illustrated in grey, starting from the end of the ORF1ab gene. Only genes containing relevant mutations are annotated. Detected mutations defining the B.1.617.2 are coloured in grey, while detected mutations not defining the B.1.617.2 lineage are coloured in red. Numbers in parentheses correspond to the number of index samples in which each mutation could be detected. (**b**) The phylogenetic tree of sequences from all seven indexes, in relation to 75% of all positive cases during this timeframe (*n* = 230). Scale bar: substitutions per site. The tree was rooted to a SARS-CoV-2 Pango lineage B.1.160. Individual sequences are denoted by terminal nodes (black circles), and clades containing multiple sequences are denoted by grey triangles.

The SARS-CoV-2 samples from index 1–7 were sequenced as part of the regional surveillance for VOC. All seven samples (index 1–7) were designated to the Pangolin lineage B.1.617.2, and an additional mutational analysis strongly suggested that index 1–7 were all infected with a B.1.617.2 variant of SARS-CoV-2 (Figure 1a). In addition, a S:E484Q mutation was detected in the spike protein of all seven sequences. This mutation was not a defining mutation for the B.1.617.2 lineage (Table 1 and Figure 1a). Phylogenetic analysis of all sequenced SARS-CoV-2 isolates in the Uppsala County during the first two weeks of August 2021 revealed that all seven indexes clustered into a distinct clade (Figure 1b).

## 4. Discussion

We presented a flight-associated transmission event of a newly emerged combination of SARS-CoV-2 lineage B1.617.2 with an S:E484Q point mutation by merging clinical contact tracing with molecular epidemiology. The emergence of the S:E484Q mutation in B1.617.2 is of particular concern, since it might provide a rapidly spreading VOC with an enhanced ability to evade antibody neutralization even in vaccinated or previously infected individuals (6). Indeed, among the index cases in this outbreak there were no differences in the intensity or type of symptoms between fully vaccinated (*n* = 3), partially vaccinated (*n* = 3), and those with unknown vaccination status (*n* = 1) (Table 2).

Whether the gain of S:E484Q affects transmissibility remains to be studied. However, we estimate the transmissibility to be high as we observed short incubation times and low cq values that could be caused by high virus titres in the samples (data not shown). This observation is further supported by a 27% increase in the total number of B.1.617.2 + S:E484Q sequences deposited in GISAID within one week (16 to 23 August 2021) (8). The acquisition of the S:E484Q mutation in the B.1.617.2 lineage was quite recent (first detected 30 January 2021) but is now rapidly spreading, likely due to selective advantage.

Among samples that were not sequenced or sequences not deposited in GISAID, there is a high risk that the occurrence of this particular lineage is underreported, since the Pangolin lineage assigner ignores the presence/absence of the S:E484Q mutation in the B.1.617.2 lineage. This concern is supported by the fact that only three cases of this combination were reported from Portugal to the end of February 2022. In contrast to this, and as a result of active surveillance regarding this specific combination, some B.1.617.2 + S:E484Q isolates were detected in patients through December 2021 in Sweden.

Hence, we propose that sequences of the B.1.617.2 lineage should be actively surveyed for the presence of the S:E484Q mutation. Furthermore, the rapid spread of this variant together with its ability to cause disease in vaccinated individuals should prompt more active contact tracing using the test, trace, and isolate regimen.

A limitation in our study is that the secondary attack rate could not be properly assessed as access to passenger lists was unavailable.

In addition to combating the pandemic with effective vaccines, additional measures such as test, trace, and isolate must be implemented to be vigilant to minimize the risk of vaccine-resistant strains taking over. In the situation described, a spreading event is most likely to have occurred during flight even though all precautions of maintaining distance and wearing facial masks were correctly performed. The vaccine status of the infected people was as recommended at this time. However, even double-vaccinated individuals were infected, had serious symptoms, and spread the virus. The vaccine efficacy in preventing Delta infection differs depending on time since last dose, the human predisposition, and the virus lineage.

The scenario shows the high level of contagiousness of specific SARS-CoV-2 combinations and the need for specific measures for different virus lineages. One additional measure of prevention could have been a requirement of a negative PCR test prior to boarding.

## 5. Conclusions

We described the emergence of a new SARS-CoV-2 variant, the B.1.617.2 lineage with the S:E484Q mutation, and its rapid spread and immune escape in vaccinated individuals. The uneven rate of the global vaccination campaign likely promotes biological selection with immune escape variants. Hence, vaccination without any additional measures may have a short-lived effect terminated by the emergence of immune escape variants as described here. Intensified genomic surveillance combined with clinical contact tracing is urged to limit the spread of such variants.

## Figures and Tables

**Table 1:** Mutations of the investigated sequences.

Index	Gisaid Acc. No.	Defining Mutations for AY.9.2; 1			Defining Mutations for B.1.617.2														Additional Mutations	
		S:A222V	S:D614G	N:D63G	S:E156G	S:D950N	S:F157del	S:L452R	S:P681R	S:R158del	S:T19R	S:T478K	M:I82T	N:D377Y	N:R203M	orf3 S26L	orf7a T120I	orf7a V82A	S:S943P	S:E484Q
**1**	EPI_ISL_3504463	1	1	0	1	1*	1	1	1	1	1	1	1	1	1	1	1	1	1	1
**2**	EPI_ISL_3504513	1	1	0	1	1*	1	1	1	1	1	1	1	1	1*	1	1	1	1	1
**3**	EPI_ISL_3504510	N	1	0	N	N	N	1	1	N	1	1	1	1	N	1	1	1	1	1
**4**	EPI_ISL_3504453	1	1	0	1	1	1	1	1	1	1	1	1	1	1	1	1	1	0	1
**5**	EPI_ISL_3665574	N	1	0	N	1	N	1	1	N	1	1	1	N	1	1	1	1	N	1
**6**	EPI_ISL_3665603	1	1	0	1	0	1	1	1	1	1	1	1	1	1	1	1	1	1	1
**7**	EPI_ISL_3665586	1	1	0	1*	N	1*	1	1	1*	1	1	1	1	1	1	1	1	1	1

1: mutation present; 1*: mutation present (confirmed by resequencing); N: N stretches; 0: mutation not present.

**Table 2:** Information about all index cases. Onset (days post-flight) applies to the Faro flight for index 1-4 and the Malaga flight for index 6 and 7.

Index	Sex	Age	Onset (Days Post-Flight)	Symptoms	Vaccine (Doses)	Date for Latest Vaccine Dose	Facial Mask	Relation
**1**	M	35	1	1, 2, 5, 7, 9, 10	PfB (1)	24 June	Yes	Couple
**2**	F	33	2	1, 2, 5, 7, 9, 10	PfB (1)	28 June	Yes	Couple
**3**	M	54	3	1, 2, 3, 5, 7, 8	PfB (2)	22 June	Yes	Cabin crew member
**4**	F	30	2	1, 2, 5	PfB (1)	4 August	Yes	Customs officer
**5**	F	56	6 *	1, 2, 3, 5, 7	PfB (2)	22 June	No	Cabin crew member’s wife
**6**	F	33	2	1, 2, 3, 4, 5, 6,7	PfB (2)	3 March	Yes	Malaga traveler
**7**	F	43	6	N/A	N/A	N/A	Yes	Malaga traveler

PfB: Pfizer Inc. and BioNTech (BNT162b2). Symptoms: fever (1), headache (2), retro orbital pain (3), spinning vertigo (4), myalgia (5), dyspnoea (6), anosmia (7) stomach pain (8) sore throat (9) cough (10). * Onset of symptoms after index 3 returned home. N/A = Not Available.

## Data Availability

The sequences are deposited in the Gisaid database (see Table 1 and Figure 1b). Further data are available upon request.
